# Supplementary material for: Expression of pH-Sensitive TRPC4 in Common Skin Tumors
Source: Int J Mol Sci. 2023 Jan 5;24(2):1037. doi: 10.3390/ijms24021037 (PMC9862651; doi:10.3390/ijms24021037)
Supplement: Supplementary file 1 [file ijms-24-01037-s001.zip › ijms-2064638-supplementary.pdf]

**SUPPLEMENTARY INFORMATION**

**1**

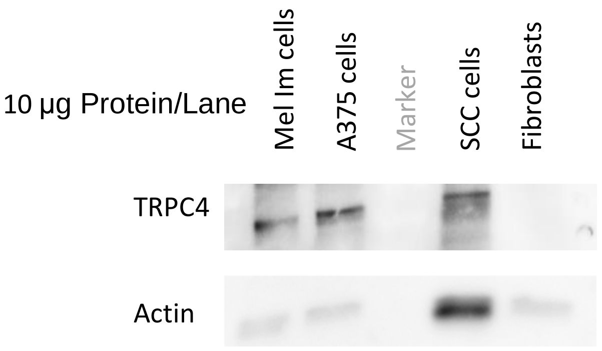


**2**

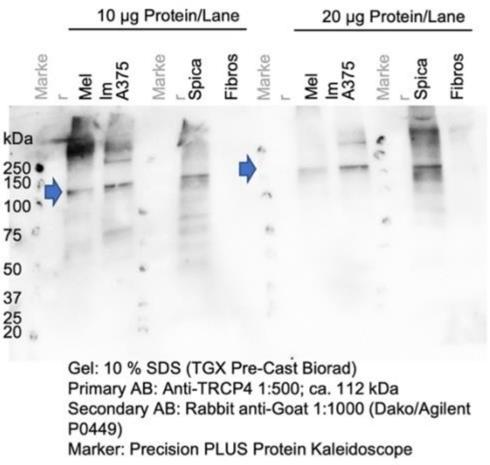


**3**

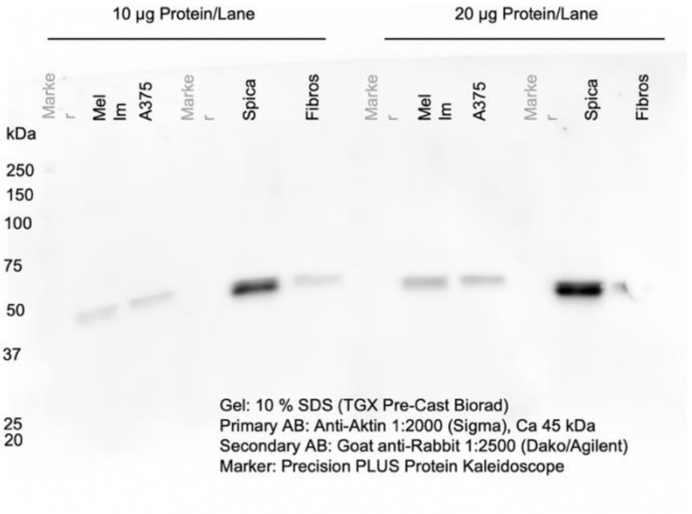


**Supplementary Figure S1: Western Blot of TRPC4 antibody.**

**1** Determination of the specificity of the TRPC 4 antibody by western blot at a concentration of 10 µg per lane in Mel Im cells, A 375 cells, cells of squamous cell carcinoma (SCC), fibroblasts, in comparison to the housekeeper actin.

**2, 3** Determination of the specificity of the TRPC 4 antibody by western blot at a concentration of 10 µg and 20 per lane in Mel Im cells, A 375 cells, cells of squamous cell carcinoma (SCC), fibroblasts, in comparison to the housekeeper actin.

**Supplementary Figure S2-S4:** Immunohistochemistry for TRPC4 in BCC. Scale bars represent 200 µm. In BCC, TRPC4 expression was weak positive for 16/39 samples (1, 3, 6, 8, 14, 15, 18, 19, 23, 24, 25, 30, 31, 33, 34, 35). 20/39 (2, 4, 5, 7, 9-13, 16, 17, 20, 26-28, 32, 36-39) showed a negative staining. 3/39 showed a strong positive reaction (21, 22, 29).

1 = A1M_1S_1, 2 = A1M_2S_1, 3 = A1M_3S_1, 4 = A1M_4S_1, 5 = A1M_5S_1, 6 = A1M_6S_1, 7 = A1M_7S_1, 8 = A1M_8S_1, 9 = A1M_9S_1, 10 = A1M_10S_1, 11 = , A1M_11S_1, 12 = A1M_12S_1, 13= A1M_16S_1, 14= A1M_17S_1, 15= A1M_18S_1, 16= A2M_1S_1, 17= A2M_3S_1, 18= A2M_4S_1, 19= A2M_5S_1, 20= A2M_6S_1, 21= A2M_9S_1, 22= A2M_11S_1, 23= A2M_12S_1, 24= A2M_13S_1, 25= A2M_14S_1, 26= A2M_18S_1, 27= A2M_20S_1, 28= A3M_1S_1, 29= A3M_3S_1, 30= A3M_4S_1, 31= A3M_7S_1, 32= A3M_10S_1, 33= A3M_11S_1, 34= A3M_12S_1, 35= A3M_13S_1, 36= A3M_14S_1, 37= A3M_15S_1, 38= A3M_16S_1, 39= A3M_17S_1

**Supplementary Figure S2:** Immunohistochemistry for TRPC4 in BCC (part 1)

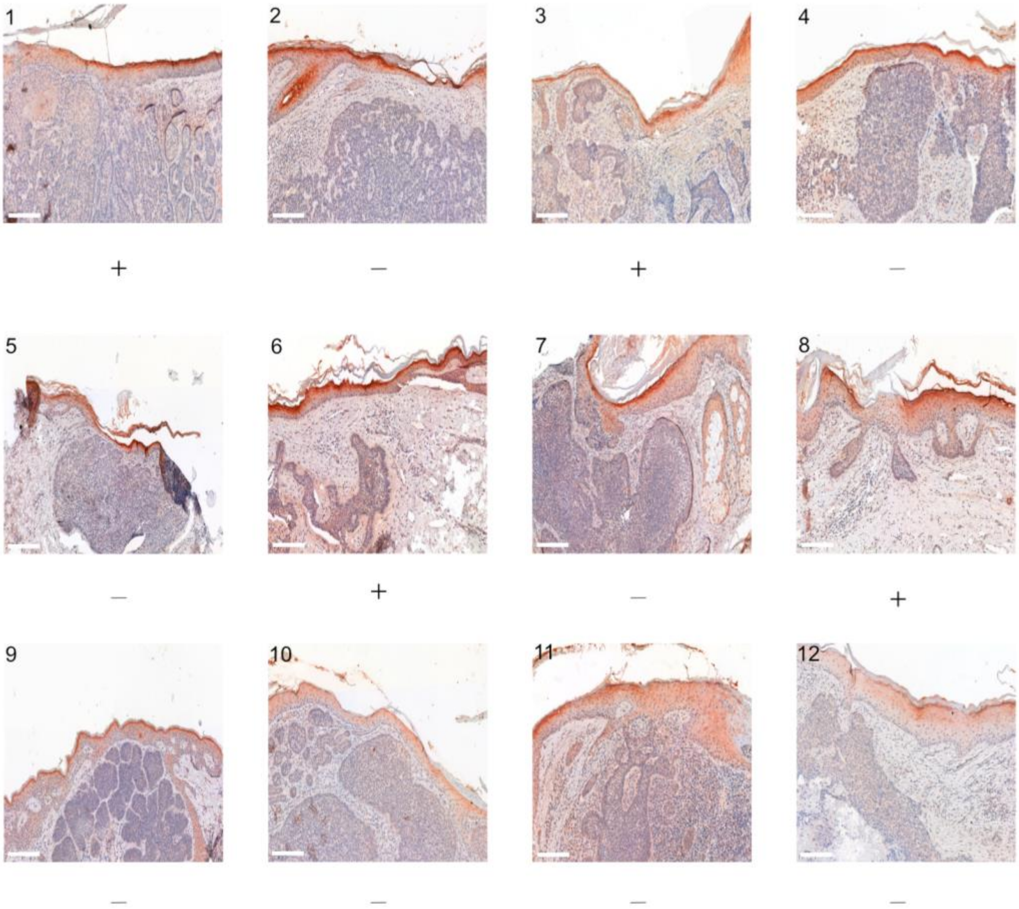

**Supplementary Figure S3:** Immunohistochemistry for TRPC4 in BCC (part 2)

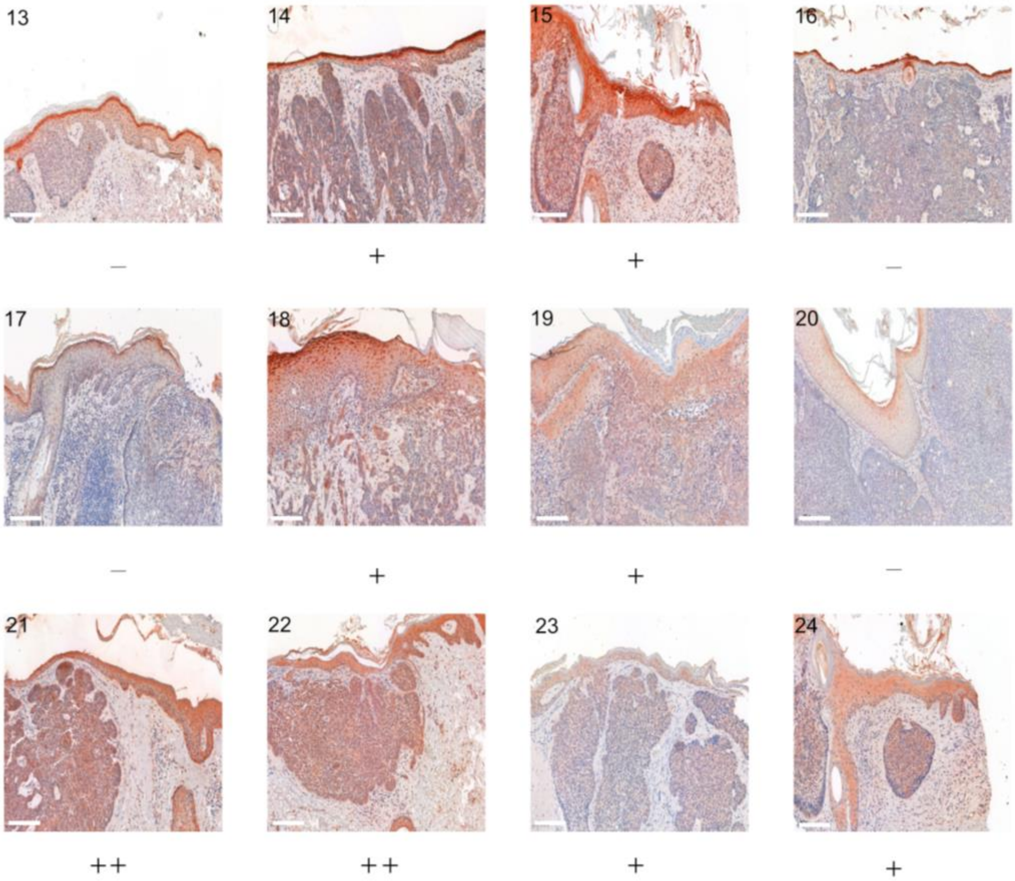

**Supplementary Figure S4:** Immunohistochemistry for TRPC4 in BCC (part 3)

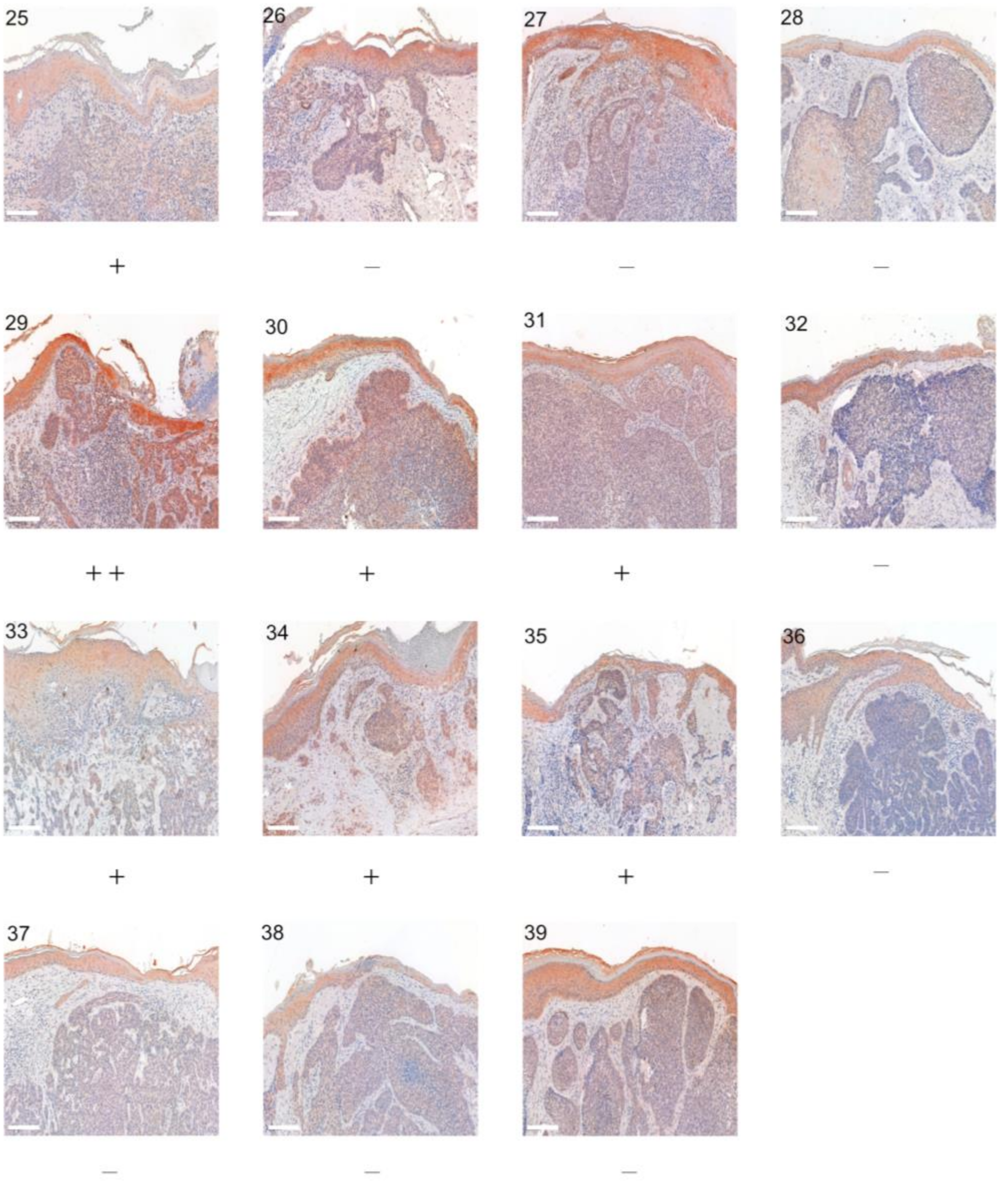

**Supplementary Figure S5-S7:** Immunohistochemistry for TRPC4 in SCC. Scale bars represent 200 µm. SCC showed a rather homogeneous expression in all 25 samples (1-27), displaying a weak positive reaction.

1= A3M_19S_1, 2= A3M_20S_1, 3= A4M_1S_1, 4= A4M_3S_1, 5= A4M_4S_1, 6= A4M_7S_1, 7= A4M_8S_1, 8= A4M_10S_1, 9= A4M_12S_1, 10= A4M_15S_1, 11= A4M_16S_1, 12= A4M_17S_1, 13= A4M_18S_1, 14= A4M_19S_1, 15= A5M_1S_1, 16= A5M_2S_1, 17= A5M_5S_1, 18= A5M_6S_1, 19= A5M_9S_1, 20= A5M_11S_1, 21= A5M_12S_1, 22= A5M_14S_1, 23= A5M_16S_1, 24= A5M_17S_1, 25= A5M_18S_1, 26= A5M_19S_1, 27= A5M_20S_1

**Supplementary Figure S5** Immunohistochemistry for TRPC4 in SCC (part 1)

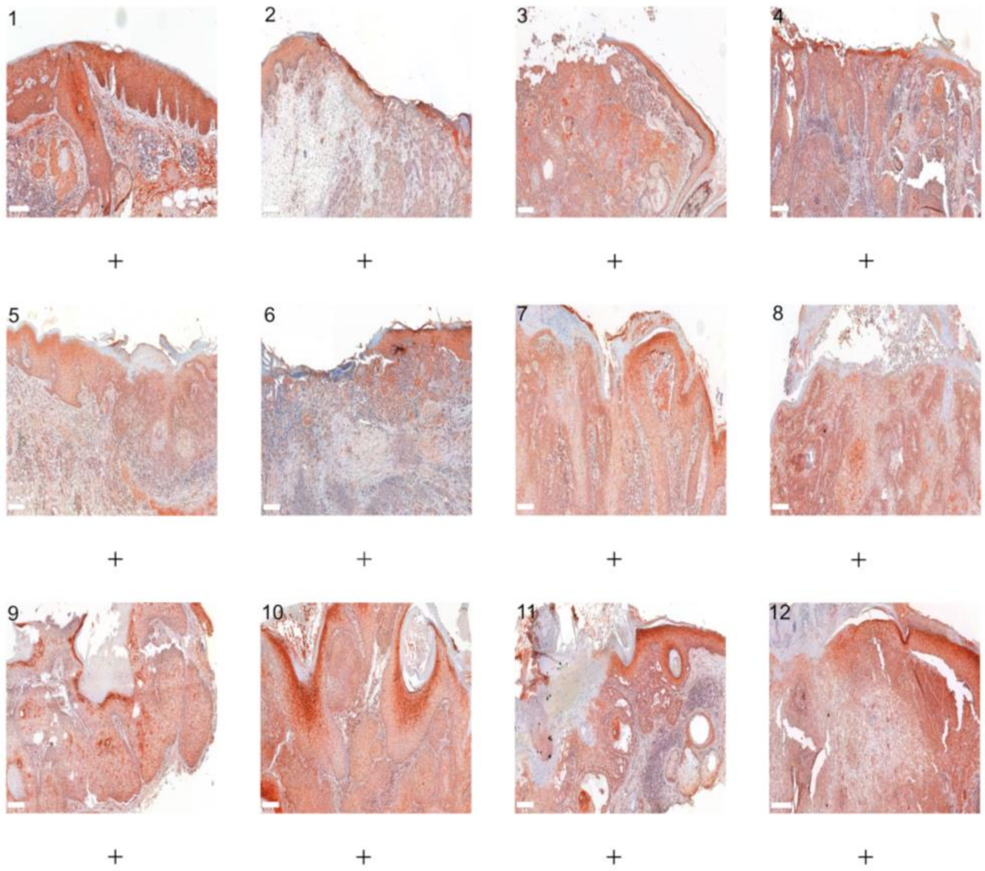

**Supplementary Figure S6** Immunohistochemistry for TRPC4 in SCC (part 2)

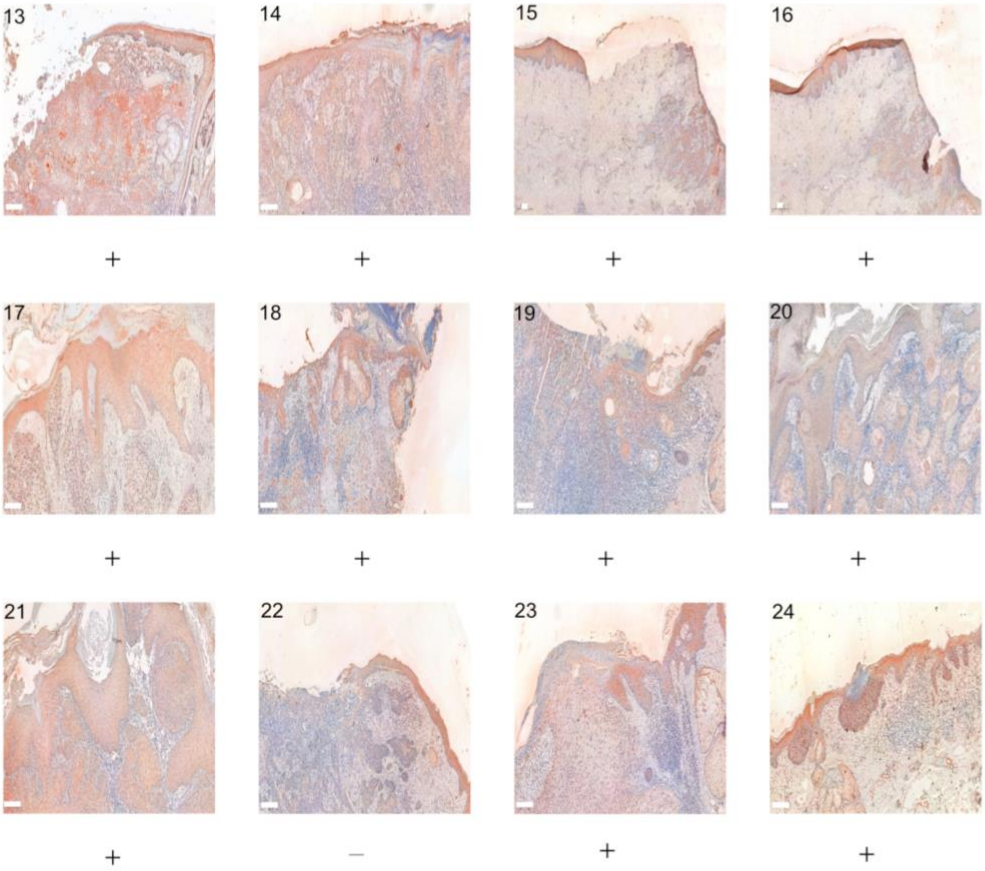


**Supplementary Figure S7** Immunohistochemistry for TRPC4 in SCC (part 3)

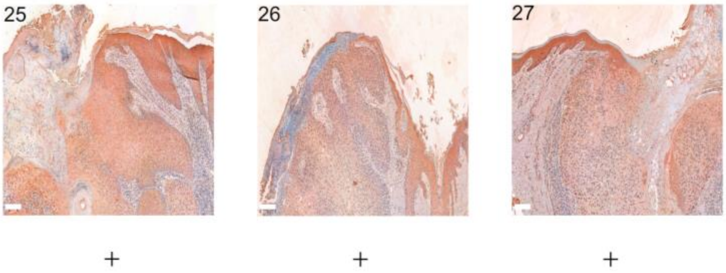

**Supplementary Figure S8:** Immunohistochemistry for TRPC4 in NCN. Scale bars represent 200 µm. In epidermal parts, 10/13 showed a weak positive reaction for TRPC4 (2, 3, 4, 5, 6, 8, 10, 12, 13, 15), 1/13 reacted strong positive (9), and 2/13 (7,14) displayed a weak staining. The dermal portion showed a weak positive expression of TRPC4 in 11/13 (2, 3, 4, 5, 6, 8, 9, 10, 11, 12, 13). For number 7 and 13, a decreasing expression towards deeper tissue levels can be observed.

1= NZN TRPC4 27-11, 2= NZN TRPC4 30-11, 3= NZN TRPC4 30-11_24-9-2021_16-00-41, 4= NZN TRPC4 41-11, 5= NZN TRPC4 101-11, 6= NZN TPC4 109-11 2, 7= K. NZN TRPC4 117-11, 8= K. NZN TRPC4 121-11, 9= K. NZN TRPC4 122-11, 10= K. NZN TRPC4 265-11, 11= K. NZN TRPC4 496- 11, 12= K. NZN TRPC4 1496- 11, 13= K. NZN TRPC4 9629- 09, 14= K. NZN TRPC4 16892- 09, 15= K. NZN TRPC4 29215- 0

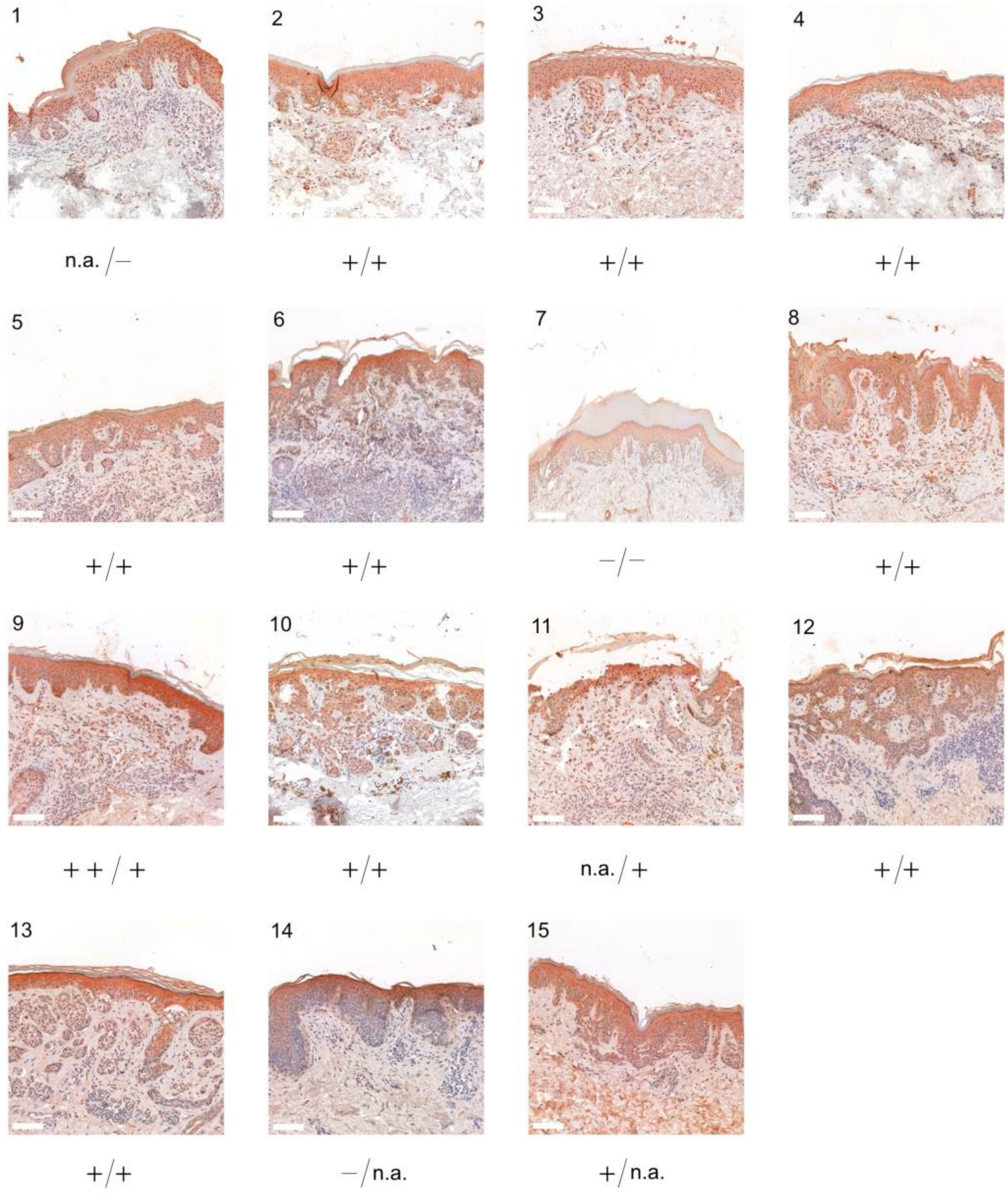

**Supplementary Figure S9:** Immunohistochemistry for TRPC4 in MM. Scale bars represent 200 µm. 12/ 13 of the epidermal and dermal sections were weak positive for TRPC4 (1-12) , whereas 1/13 of showed a strong positive epidermal and negative dermal staining reaction (13).

1= MM TRPC4 1043-11, 2= MM TRPC4 1730- 11, 3= MM TRPC4 1903-11, 4= MM TRPC4 1944-11, 5= MM TRPC4 2013-11, 6= MM TRPC4 2017-11, 7= MM TRPC4 2125- 11, 8= MM TRPC4 2668- 09, 9= MM TRPC4 7060- 09, 10= MM TRPC4 7104- 09, 11= MM TRPC4 7677- 09, 12= MM TRPC4 7802- 09, 13= MM TRPC4 7439-09

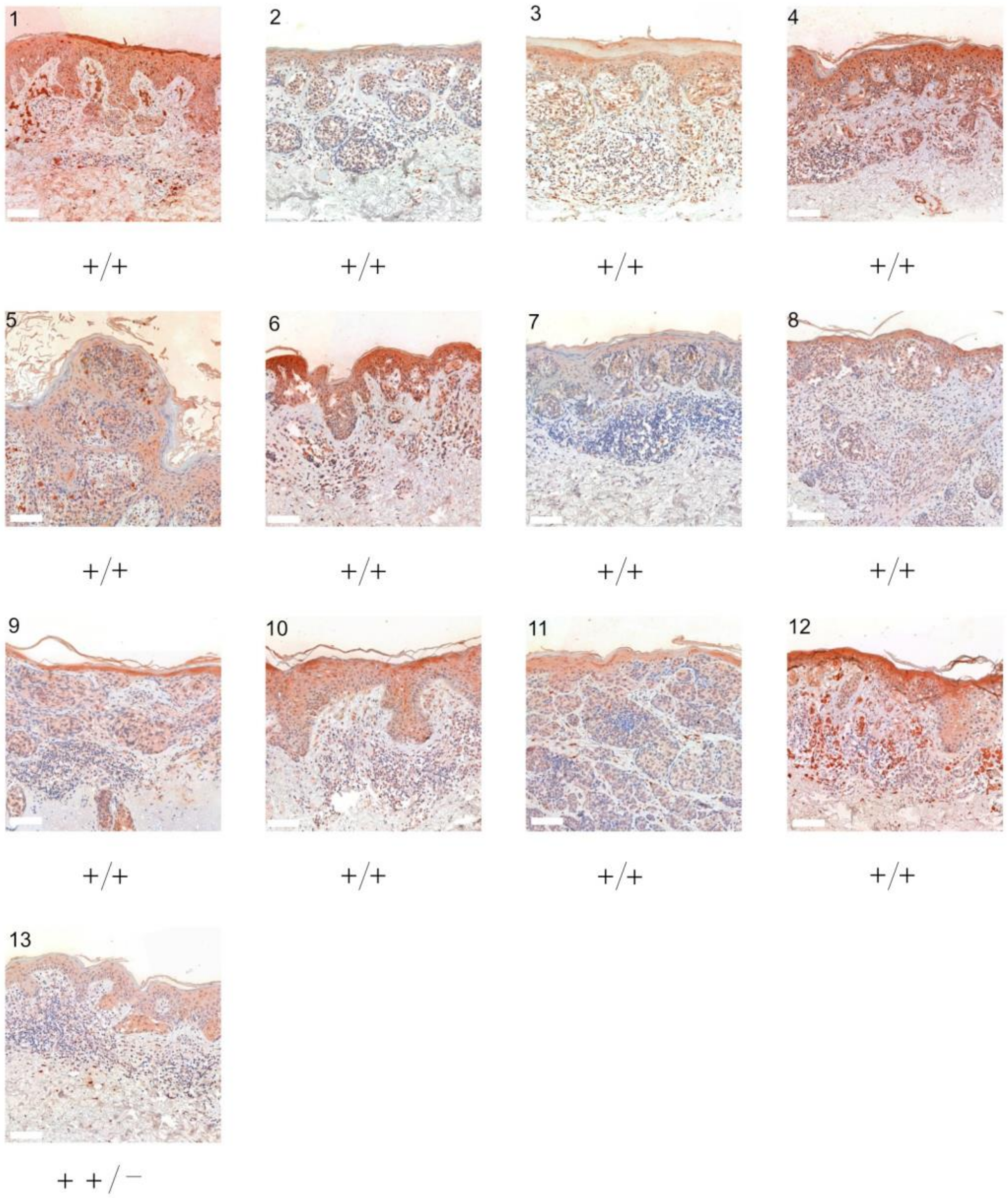

**Supplementary Figure S10:** TRPC4 mutation frequency in MM from cBioportal.org

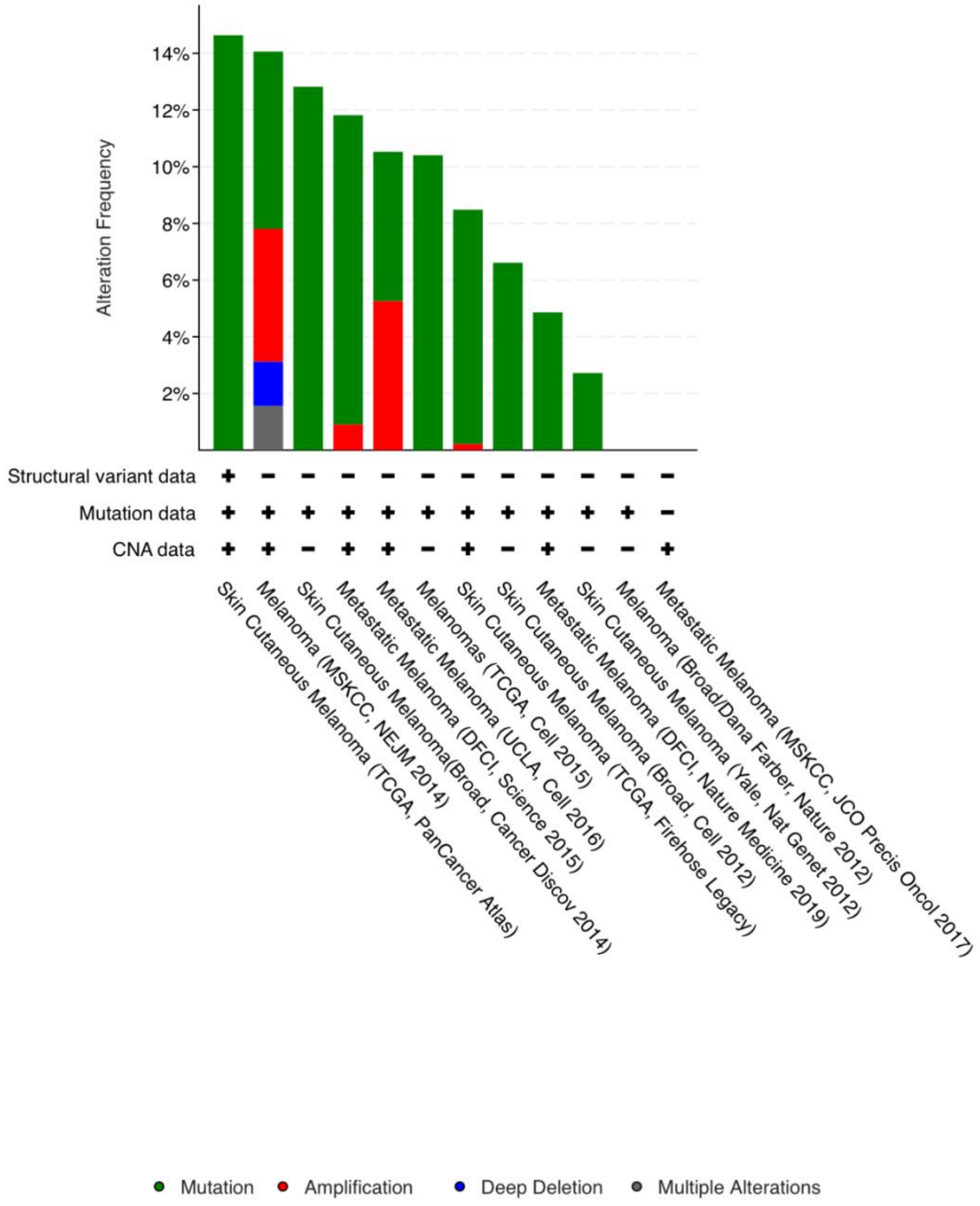

**Supplementary Figure S11:** TRPC 4 Mutation frequency in non-melanoma skin cancer from cBioportal.org

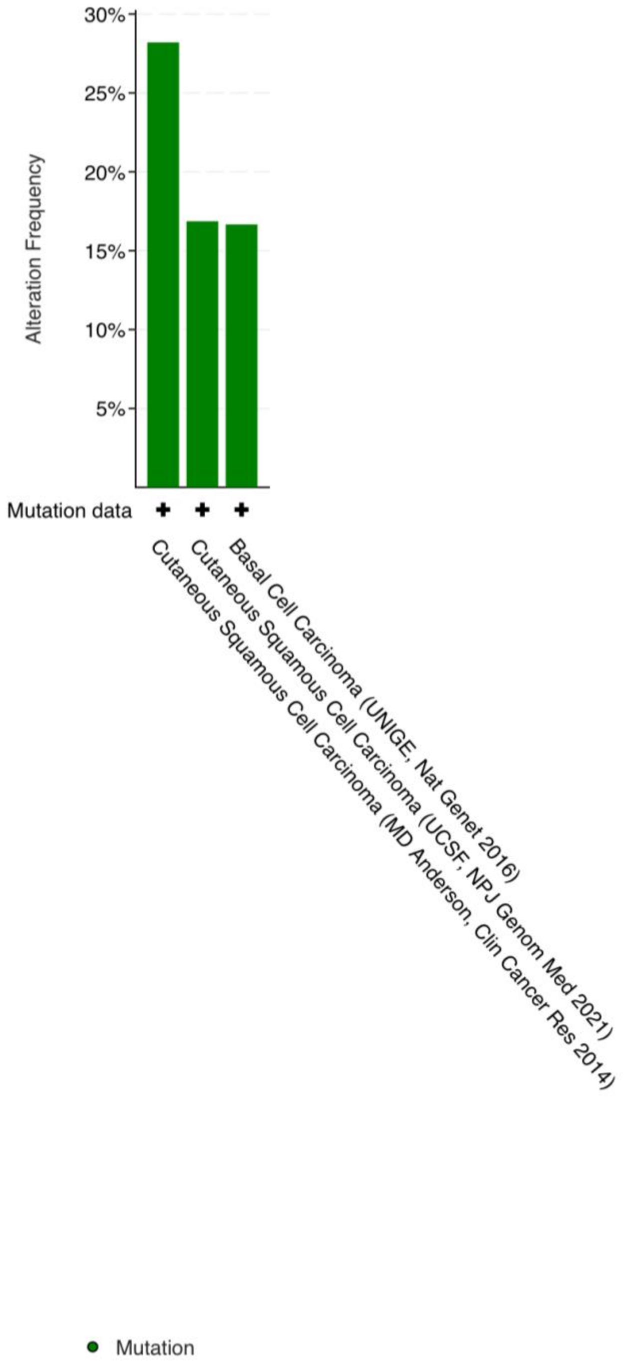

**Supplementary Table S1:** Scoring results for TRPC4 in BCC

| Number | Type of BCC | Scoring |
|---|---|---|
| A1M_1S_1 | nodular | + |
| A1M_2S_1 | nodular | - |
| A1M_3S_1 | nodular | + |
| A1M_4S_1 | nodular | - |
| A1M_6S_1 | nodular | - |
| A1M_7S_1 | nodular | + |
| A1M_8S_1 | nodular | - |
| A1M_9S_1 | superficial | + |
| A1M_11S_1 | nodular | - |
| A1M_12S_1 | nodular | - |
| A1M_13S_1 | nodular | - |
| A1M_15S_1 | nodular | - |
| A1M_16S_1 | superficial | - |
| A1M_17S_1 | nodular | + |
| A1M_18S_1 | superficial | + |
| A2M_1S_1 | nodular | - |
| A2M_3S_1 | nodular | - |
| A2M_4S_1 | sclerosing | + |
| A2M_5S_1 | nodular / sclerosing (mixed) | + |
| A2M_6S_1 | nodular | - |
| A2M_9S_1 | nodular | ++ |
| A2M_11S_1 | nodular | ++ |
| A2M_12S_1 | nodular | + |
| A2M_13S_1 | nodular | + |
| A2M_14S_1 | nodular / sclerosing (mixed) | + |
| A2M_18S_1 | nodular / sclerosing (mixed) | - |
| A2M_20S_1 | nodular | - |
| A3M_1S_1 | nodular | - |
| A3M_3S_1 | nodular | ++ |
| A3M_4S_1 | nodular | + |
| A3M_7S_1 | nodular | + |
| A3M_10S_1 | nodular | - |
| A3M_11S_1 | nodular / sclerosing (mixed) | + |
| A3M_12S_1 | nodular / sclerosing (mixed) | + |
| A3M_13S_1 | nodular / sclerosing (mixed) | + |
| A3M_14S_1 | nodular | - |
| A3M_15S_1 | nodular | - |
| A3M_16S_1 | nodular | - |
| A3M_17S_1 | nodular | - |

**Supplementary Table S2:** Scoring results for TRPC4 in SCC

| Number | Scoring |
|---|---|
| A3M_19S_1 | + |
| A3M_20S_1 | + |
| A4M_1S_1 | + |
| A4M_3S_1 | + |
| A4M_4S_1 | + |
| A4M_7S_1 | + |
| A4M_8S_1 | + |
| A4M_10S_1 | + |
| A4M_12S_1 | + |
| A4M_15S_1 | + |
| A4M_16S_1 | + |
| A4M_17S_1 | + |
| A4M_18S_1 | + |
| A4M_19S_1 | + |
| A5M_1S_1 | + |
| A5M_2S_1 | + |
| A5M_5S_1 | + |
| A5M_6S_1 | + |
| A5M_9S_1 | + |
| A5M_11S_1 | + |
| A5M_12S_1 | + |
| A5M_14S_1 | - |
| A5M_16S_1 | + |
| A5M_17S_1 | + |
| A5M_18S_1 | + |
| A5M_19S_1 | + |
| A5M_20S_1 | + |

**Supplementary Table S3:** Scoring results for TRPC4 in NCN

| Number | Type of NCN | Scoring Epidermal | Scoring Dermal |
|---|---|---|---|
| NZN TRPC4 27-11 | dermal | n/a | - |
| NZN TRPC4 30-11 | compound | + | + |
| NZN TRPC4 30-11_24-9-2021_ 16-00-41 | compound | + | + |
| NZN TRPC4 41-11 | compound | + | + |
| NZN TRPC4 101-11 | compound | + | + |
| NZN TPC4 109-11 2 | compound | + | + |
| K. NZN TRPC4 117-11 | compound | - | - |
| K. NZN TRPC4 121-11 | compound | + | + |
| K. NZN TRPC4 122-11 | compound | ++ | + |
| K. NZN TRPC4 265-11 | compound | + | + |
| K. NZN TRPC4 496- 11 | dermal | n/a | + |
| K. NZN TRPC4 1496- 11 | compound | + | + |
| K. NZN TRPC4 9629- 09 | compound | + | + |
| K. NZN TRPC4 16892- 09 | junctional | - | n/a |
| K. NZN TRPC4 29215- 09 | junctional | + | n/a |

**Supplementary Table S4:** Scoring results for TRPC4 in MM

| Number | Scoring epidermal | Scoring dermal |
|---|---|---|
| MM TRPC4 1043-11 | + | + |
| MM TRPC4 1730- 11 | + | + |
| MM TRPC4 1903-11 | + | + |
| MM TRPC4 1944-11 | + | + |
| MM TRPC4 2013-11 | + | + |
| MM TRPC4 2017-11 | + | + |
| MM TRPC4 2125- 11 | + | + |
| MM TRPC4 2668- 09 | + | + |
| MM TRPC4 7060- 09 | + | + |
| MM TRPC4 7104- 09 | + | + |
| MM TRPC4 7677- 09 | + | + |
| MM TRPC4 7802- 09 | + | + |
| MM TRPC4 7439-09 | ++ | - |

**Supplementary Table S5:** Statistical analysis of TRPC4– comparison of all entities

| | | Scoring | | | total |
|---|---|---|---|---|---|
| | | - | + | ++ | |
| SCC | number | 0 | 25 | 0 | 25 |
| | % of tumors | 0% | 100.0% | 0% | 100% |
| BCC | number | 20 | 16 | 3 | 39 |
| | % of tumors | 51.3% | 41.0% | 7.7% | 100% |
| NCN epidermal | number | 2 | 10 | 1 | 13 |
| | % of tumors | 15.4% | 76.9% | 7.7% | 100% |
| NCN dermal | number | 2 | 11 | 0 | 13 |
| | % of tumors | 15.4% | 84.6% | 0% | 100% |
| MM epidermal | number | 0 | 12 | 1 | 13 |
| | % of tumors | 0% | 92.3% | 7.7% | 100% |
| MM dermal | number | 1 | 12 | 0 | 13 |
| | % of tumors | 7.7% | 92.3% | 0% | 100% |
| Gesamt | number | 25 | 86 | 5 | 116 |
| | % of tumors | 21.6% | 74.1% | 4.3% | 100% |

**Supplementary Table S6:** NCBI Geo gene expression analysis of TRPC4

BCC vs. SCC: Study GSE53462

| ID | adj.P.Val | P.Value | t | B | logFC | Gene.symbol | Gene.title |
|---|---|---|---|---|---|---|---|
| **ILMN_1782295** | 0.945847 | 8.35e-01 | -0.2115083 | -6.17258 | -0.01273726 | TRPC4 | transient receptor potential cation channel subfamily C member 4 |

NCN cs. MM: Study GSE4587

| ID | adj.P.Val | P.Value | t | B | logFC | Gene.symbol | Gene.title |
|---|---|---|---|---|---|---|---|
| **224220_x_at** | 0.2202 | 0.04481366 | -2.2047613 | -3.9612 | -1.3656916 | TRPC4 | transient receptor potential cation channel subfamily C member 4 |
| **220818_s_at** | 0.5408 | 0.27021535 | -1.1482785 | -5.41951 | -0.8524036 | TRPC4 | transient receptor potential cation channel subfamily C member 4 |
| **224219_s_at** | 0.9163 | 0.80658083 | 0.2495519 | -6.02526 | 0.2283259 | TRPC4 | transient receptor potential cation channel subfamily C member 4 |
| **220817_at** | 581 | 0.31098903 | -1.051415 | -5.51844 | -0.8847788 | TRPC4 | transient receptor potential cation channel subfamily C member 4 |

NCN cs. MM: Study GSE12391

| ID | adj.P.Val | P.Value | t | B | logFC | Gene.symbol | Gene.title |
|---|---|---|---|---|---|---|---|
| **19602** | 6.09e-01 | 3.72e-01 | 0.8958235 | -6.0441039 | 0.01984097 | TRPC4 | transient receptor potential cation channel subfamily C member 4 |

NCN cs. MM: Study GSE46517

| ID | adj.P.Val | P.Value | t | B | logFC | Gene.symbol | Gene.title |
|---|---|---|---|---|---|---|---|
| **220817_at** | 6.26e-01 | 3.18e-01 | 1.010048 | -5.803297 | 0.3355734 | TRPC4 | transient receptor potential cation channel subfamily C member 4 |
| **220818_s_at** | 9.58e-01 | 8.75e-01 | 0.158867 | -6.289573 | 0.0550281 | TRPC4 | transient receptor potential cation channel subfamily C member 4 |

NCN cs. MM: Study GSE114445

| ID | adj.P.Val | P.Value | t | B | logFC | Gene.symbol | Gene.title |
|---|---|---|---|---|---|---|---|
| **220818_s_at** | 0.25245972 | 7.80e-02 | -1.828659 | -4.7264186 | -0.11600316 | TRPC4 | transient receptor potential cation channel subfamily C member 4 |
| **224220_x_at** | 0.25438021 | 7.90e-02 | -1.822226 | -4.7368991 | -0.13412296 | TRPC4 | transient receptor potential cation channel subfamily C member 4 |
| **220817_at** | 0.30558838 | 1.09e-01 | -1.656632 | -4.9965644 | -0.10672587 | TRPC4 | transient receptor potential cation channel subfamily C member 4 |

NCN cs. MM: Study GSE183115

| ID | adj.P.Val | P.Value | t | B | logFC | Gene.symbol | Gene.title |
|---|---|---|---|---|---|---|---|
| **ADXECADA.22327_s_at** | 919 | 0.29257616 | 1.11 | -5.20838 | 1.23e-01 | TRPC4 | transient receptor potential cation channel subfamily C member 4 |
| **ADXECNTDJ.362_s_at** | 963 | 0.58561657 | -5.64e-01 | -5.6313 | -5.93e-02 | TRPC4 | transient receptor potential cation channel subfamily C member 4 |
